# *Koelreuteria paniculata* Seed Oil—A Rich Natural Source of Unsaturated Fatty Acids and Phytocompounds with DNA Protective Potential

**DOI:** 10.3390/foods12112230

**Published:** 2023-06-01

**Authors:** Tsvetelina Andonova, Yordan Muhovski, Elena Apostolova, Samir Naimov, Zhana Petkova, Olga Teneva, Ginka Antova, Iliya Slavov, Ivanka Dimitrova-Dyulgerova

**Affiliations:** 1 Department of Botany and Biological Education, Faculty of Biology, University of Plovdiv “Paisii Hilendarski”, 24 Tzar Asen Street, 4000 Plovdiv, Bulgaria; ts_andonova@uni-plovdiv.bg (T.A.); ivadim@uni-plovdiv.bg (I.D.-D.); 2 Life Sciences Department, Walloon Agricultural Research Centre, 5030 Gembloux, Belgium; 3 Department of Plant Physiology and Molecular Biology, Faculty of Biology, University of Plovdiv “Paisii Hilendarski”, 24 Tzar Asen Street, 4000 Plovdiv, Bulgaria; eapostolova@uni-plovdiv.bg (E.A.); naimov0@uni-plovdiv.bg (S.N.); 4 Department of Chemical Technology, University of Plovdiv “Paisii Hilendarski”, 24 Tzar Asen Street, 4000 Plovdiv, Bulgaria; zhanapetkova@uni-plovdiv.bg (Z.P.); olga@uni-plovdiv.bg (O.T.); ginant@uni-plovdiv.bg (G.A.); 5 Department of Biology, Faculty of Pharmacy, Medical University of Varna, 9000 Varna, Bulgaria; ijelev80@abv.bg

**Keywords:** *Koelreuteria paniculata*, seed oil, chemical composition, physicochemical characteristics, cytotoxic activity, DNA protection

## Abstract

The present work is focused on the physicochemical characteristics, chemical composition, and some biological activities of *Koelreuteria paniculata* seed oil. The glyceride oil, obtained with a Soxhlet apparatus by extraction with hexane, was characterized by a relatively high oil content (over 20%), and it is defined as a non-drying oil (iodine value—44 gI_2_/100 g) with good oxidative stability (over 50 h). There were identified 11 fatty acids, 6 sterols, 3 tocopherols, and 6 phospholipids, as the last group was reported for the first time. The major components among them were—monounsaturated eicosenoic and oleic acids, *β*-sitosterol, *β*-tocopherol, and phosphatidylcholine. The in vitro tests demonstrated DNA protective activity and a lack of cytotoxicity of the oil, data that has been reported for the first time. The in vitro MTT test of the oil on HT-29 and PC3 cell lines did not indicate antitumor activity. The seed oil studied contains valuable bio-components, which have proven benefits for human health, and that is why it could be used in food, cosmetic, and pharmaceutical products.

## 1. Introduction

Natural active ingredients, contained in plant extracts, essential and glyceride oils, and other plant products, are of increasing interest to the scientific community given their beneficial effects on human health and for a healthy lifestyle [1,2,3]. Different classes of primary and secondary metabolites, such as unsaturated fatty acids, polyphenols, flavonoids, phenolic acids, and others—responsible for the antioxidant potential of plants have been identified [2,3]. These phytochemicals, through various mechanisms, help to alleviate oxidative stress and prevent damage from ROS (reactive oxygen species)-DNA damage, lipid peroxidation, protein oxidation, etc., which are the root cause of the occurrence of various human diseases (cancer, diabetes, digestive and neurodegenerative disorders, autoimmune pathologies, etc., [4,5,6]. Unsaturated fatty acids are known to have a proven beneficial effect on the human body. A positive correlation between the consumption of essential fatty acids and the prevention of heart disease, hypertension, inflammation, cancer, neurological diseases, etc., has also been proven [3]. These valuable food compounds can be obtained from both various animal and vegetable oils [7,8].

A possible source of bioactive components with antioxidant and other beneficial properties could be the Asian tree species *Koelreuteria paniculata* Laxm. (“Golden rain tree”) from the Sapindaceae family. The species was brought to Europe for decorative purposes, but it spread rapidly, and in Bulgaria, it is considered potentially invasive [9]. The plant species is used in traditional Chinese medicine, and nowadays it is the subject of phytochemical and biological studies. The content of primary metabolites, including sugars, organic acids, amino acids, fatty acids and their esters, as well as various secondary metabolites (polyphenols, tannins, flavonoids, phenolic acids, terpenes, terpenoids, sterols, saponins, etc.), has been proven mainly in extracts and their fractions from different plant parts [10,11,12,13,14,15,16].

The compounds contained in *K. paniculata* are the basis for determination of the biological activities. The gene-protective potential of the leaf extracts and their fractions (chloroform, ethyl acetate, hexane, butanol), by applying molecular genetic methods, has been proven [4,17,18,19]. The DNA-protective capacity of the flower, leaf, and stem bark ethanolic extracts against oxidative damage were proved by [20]. Antioxidant activities of *K. paniculata* have been reported for different fractions of leaf extracts [4,17,18], carotenoid fraction of flowers [21], and extracts from aerial plant parts [20]. Antiproliferative activity of *K. paniculata* extracts and carotenoid fraction against HT-29 (colorectal adenocarcinoma), HepG2 (human hepatocarcinoma), MDA-MB-231 (human breast cancer cells), and PC3 (prostate carcinoma) tumor cell lines were also reported [21,22]. 

Relatively less research is done on the glyceride oil of golden rain tree seeds. Evaluation of the nutritional value of the seeds characterizes it as a source of oil with high oil, protein, and fiber contents [23]. A high oil content (28–30%) and a low percentage of free fatty acids (0.91%) were found by [24]. Monounsaturated, polyunsaturated, and saturated fatty acids are included in the composition of the seed oil of *K. paniculata*, among which are cis 11-eicosenoic, arachidonic, palmitic, oleic, linoleic, linolenic, stearic acids [24,25,26,27]. Other authors indicate eleven fatty acids in the seed oil, with eicosenoic acid being the most abundant [28]. The species is also of interest as a crop suitable for biofuel production, given the good characteristics of the seed oil—it contains small amounts of saturated and polyunsaturated fatty acids and has desirable values of iodine number and cetane number [24,25,27,28].

The present study is a continuation of our team’s research on *K. paniculata* in search of valuable phytocomponents of useful applications. It aims to study the glyceride oil of *K. paniculata* seeds, namely its chemical composition, and physicochemical characteristics, as well as DNA protective, cytotoxic, and antitumor activities. This goal stems from the fact that there is a lack of research data on the biological activities of *K. paniculata* seed oil. 

## 2. Materials and Methods

### 2.1. Plant Material Collection and Isolation of the Glyceride Oil

**Figure 1:**
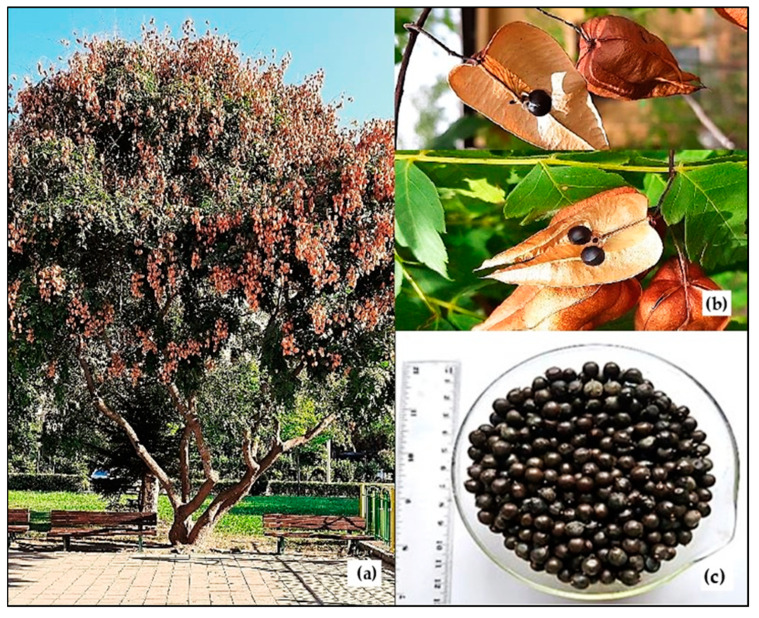
*Koelreuteria paniculata* tree (**a**), fruits (**b**), and seeds (**c**).

The seeds of *K. paniculata* were collected from Plovdiv city (N42°8′9.9492″, E24°44′31.8048″), Bulgaria, in September 2022 (Figure 1). Herbarium specimens of the tree species under No. 060436 have been deposited at the herbarium of the Agricultural University of Plovdiv, Bulgaria (SOA). The glyceride oil was extracted from well-ripened and grounded seeds, using hexane in a Soxhlet apparatus [29].

### 2.2. Chemical, Physicochemical and Chromatographic Methods

#### 2.2.1. Chemical Composition

Protein was determined using Kjeldahl apparatus (Velp Scientifica Srl, Via Stazione, Italy). Briefly, 0.5 g of seeds were weighed and subjected to mineralization for 35 min at 420 °C in the presence of H_2_SO_4_:H_2_O_2_ (2:1, *v*/*v*) and a catalyst. The solution was distillated in UDK 127 and the generated NH_3_ was absorbed in 4% solution of H_3_BO_3_ and then was titrated with 0.1 N H_2_SO_4_. The result obtained gives the content of nitrogen and the total protein is calculated using the factor of 6.25 [30]. The carbohydrate content was calculated by the formula: 100 − (weight in grams [protein + lipids + water + ash] in 100 g of dry seeds) [31]. The soluble carbohydrates were determined based on the ability of carbohydrates with a free carbonyl group to reduce Fehling′s reagent, due to the easy oxidation by Fehling’s solution of free aldehyde and ketone groups found in sugars. Reduced Cu_2_O is determined iodometrically—titration with Na_2_S_2_O_3_ [32]. The starch content was identified using the ability of monosaccharides to be oxidized with Cu (II) by a copper reagent after hydrochloric acid hydrolysis of the product. Then the determination is based on titration with Na_2_S_2_O_3_ [33]. Fibers, ash content, and moisture were determined gravimetrically according to [30].

#### 2.2.2. Physicochemical Properties

The physicochemical characteristics of the glyceride oil (iodine value, acid value, peroxide value, saponification value, refractive index, and relative density) were analyzed following the procedures by ISO [34,35,36,37,38,39]. Oxidative stability was measured on Rancimat 679 equipment (Metrohm, Herisau, Switzerland) at 100 °C [40].

#### 2.2.3. Fatty Acid Composition

The fatty acid composition of the seed oil was determined by gas chromatography (GC) [41]. About 100 mg of the oil was trans-esterified with sulfuric acid in methanol in order to obtain fatty acid methyl esters (FAMEs) [42]. Determination of FAMEs was performed on HP 5890 gas chromatograph equipped with Supelco SP^TM^–2560 Fused Silica Capillary Column (75 m × 0.18 mm × 0.14 μm (film thickness) (Supelco, Bellefonte, PA, USA) and a flame ionization detector (FID). The column temperature was from 140 °C (5 min), at 4 °C/min to 240 °C (3 min) and the injector and detector temperatures were at 250 °C. Identification was performed by comparison of the retention times with the retention time of a standard mixture of FAME (Supelco, USA 37 comp. FAME mix) which were subjected to GC analysis under identical conditions.

#### 2.2.4. Determination of Sterols

Three grams of the seed oil was saponificated with 2 N KOH and the unsaponifiables were extracted with hexane. Their content was determined gravimetrically [43]. Total sterols were isolated from the unsaponifiable matter by thin-layer chromatography (TLC) and their content was determined spectrophotometrically (at 597 nm) [44]. Sterol composition was determined on HP 5890 gas chromatograph. It was equipped with DB–5 capillary column (25 m × 0.25 mm) and FID. The temperature was from 90 °C (3 min) up to 290 °C at a rate of 15 °C/min and then up to 310 °C at a rate of 4 °C/min (10 min). The detector temperature was 320 °C, while injector temperature was 300 °C. Hydrogen was the carrier gas. Identification was confirmed by comparison of retention times with a standard mixture of sterols [45]. The determination of the sterols was performed using a calibration curve of *β*-sitosterol (the concentration ranged from 0 to 3000 μg/mL). The linear regression coefficient (R^2^) was 0.9987, the limit of detection (LOD) was 91 μg/mL, and the limit of quantification (LOQ) was 295 μg/mL.

#### 2.2.5. Determination of Tocopherols

Tocopherols were determined by high-performance liquid chromatography with a fluorescent detection (295 nm of excitement and 330 nm of emission) using Nucleosil Si 50-5 column (250 mm × 4 mm). The mobile phase was hexane: dioxane, 96:4 (*v*/*v*) and the flow rate was 1 mL/min [46]. Tocopherol content was calculated on the base of the tocopherol peak areas in the sample vs. tocopherol peak area of a standard tocopherol solution. 

#### 2.2.6. Determination of Phospholipids

The phospholipid classes were isolated by two-dimensional TLC [47]. The content was determined spectrophotometrically at 700 nm after mineralization of the phospholipid spots with a mixture of perchloric and sulfuric acid, 1:1 (*v*/*v*) [48].

### 2.3. DNA Nicking Protection Assay

#### 2.3.1. Reagents

Trolox (6-hydroxy-2,5,7,8-tetramethylchromane-2-carboxylic acid), hydrogen peroxide solution, and potassium phosphate dibasic were purchased from Sigma-Aldrich (Steinheim am Albuch, Germany); di-Potassium hydrogen phosphate and iron (II) sulfate heptahydrate were obtained from Merck (Darmstadt, Germany); TBE buffer and Agarose SPI were purchased from Duchefa (Haarlem, The Netherlands). 

#### 2.3.2. Determination of DNA Protection Activity 

The DNA protective effect of the glyceride oil was assessed using supercoiled plasmid DNA (pUC19) as it is described (with minor modifications) by [20,49]. Briefly, one microliter of the preparation was suspended in 10 µL of 12% solution of methylated cyclodextrin (CAVASOL W7 M, Wacker Chemie AG, Munich, Germany) and then added to 600 ng of pUC19 and subsequently incubated with Fenton’s reagent at 37 °C for 30 min. All reactions were carried out in a final volume of 20 µL. Ten microliters of different concentrations (25, 50, and 100 mg/mL) of Trolox and water were used as positive and negative controls, respectively. The reactions were subsequently analyzed by performing 1.0% agarose gel electrophoresis in 0.5× TBE buffer at 120 V for 1 h. The degree of DNA nicking was analyzed using the Gel Doc™ EZ Imaging system (Bio-Rad, Hercules, CA, USA).

### 2.4. In Vitro Cytotoxicity and Antiproliferative Activity Assays 

#### 2.4.1. Cell Culture Reagents and Lines

Dulbecco’s modified Eagle’s medium (DMEM), fetal bovine serum (FBS), antibiotics (penicillin and streptomycin), and Neutral Red were purchased from Sigma-Aldrich, Schnelldorf, Germany. The disposable consumables were supplied by Orange Scientific, Braine-l’Alleud, Belgium. The BALB/c 3T3 clone A31 (ATCC^®^ CCL-163^TM^)—mouse embryonic fibroblast, MCF-10A (ATCC^®^ CRL-10317™)—normal human epithelial, PC3 (ATCC^®^ CRL-1435™)—prostate carcinoma, and HT-29 (ATCC^®^ HTB-38™)—colorectal adenocarcinoma cell lines were obtained from American Type Cultures Collection (ATCC, Manassas, VA, USA).

The in vitro tests were performed to determine the cytotoxicity and antiproliferative activity of glyceride oil on cell lines. The findings were mathematically, statistically, and graphically processed, suitable for publishing in specialized scientific journals. 

#### 2.4.2. Cell Cultivation

Adherent cell lines were cultivated in DMEM medium (4.5 g/L glucose), 10% fetal bovine serum, 100 U/mL penicillin, and 0.1 mg/mL streptomycin in plastic, 25 cm^2^ by 75 cm^2^, cell culture dishes. The cells were maintained in a logarithmic growth phase at 37 °C and a 5% CO_2_ atmosphere. For the in vitro tests, the cells in an exponential growth phase, after trypsinization were brought to the required concentration and seeded in 96-well plates for cell culture. After 24 h of cultivation, under the above-mentioned conditions, the cells were treated with the test substances, according to the specific experimental setup.

#### 2.4.3. Determination of Cytotoxicity and Antiproliferative Activities

Neutral Red Uptake in vitro test (NRU-assay) is a colorimetric method for evaluating cell viability under in vitro conditions. This method is based on the ability of living cells to incorporate the Neutral Red dye into their lysosomes. The test determines in a short time the CC_50_ values (average cytotoxic concentrations) of the substances investigated, through which an initial assessment of the possible toxicity of the substance can be made and it is a real basis for calculating the starting concentration for additional in vitro analyzes and determining the initial dose, in in vivo toxicology experiments with various experimental animals.

Mouse embryonic fibroblasts (BALB/c 3T3, clone A31) were cultivated in 75 cm^2^ cell culture dishes as an adherent, monolayer cell culture, under standard cell culture conditions. Cells were seeded at the amount of 1 × 10^4^ cells/100 μL culture medium/well in 96-well cell culture plates. After that 24 h incubation followed, under standard conditions for achieving cell adhesion on the surface of the wells. The cells are then treated with a solution of the test substances in double-increasing concentrations. After 24 h of incubation, a culture medium containing Neutral Red was added. After 3 h of incubation, the wells were washed with PBS, pH 7.4, and lysing solution was added (ethanol/acetic acid/dH_2_O = 49/1/50). Optical density was measured using a TECAN microplate reader at λ = 540 nm. The percentage of cytotoxicity was calculated using the following formula:

Cytotoxicity (%) = (1 − (OD_570_ (sample)/OD_570_ (negative control)) × 100

The assay for antiproliferative activity was the same, with the difference that the cells were seeded in the amount of 1 × 10^3^ cells/100 μL culture medium/well, and the result was read after 72 h incubation with the substance under investigation.

Selective index (SI): SI = IC_50_ MCF − 10A/IC_50_ Tumor cells

### 2.5. Statistical Analysis

All chemical compounds and physicochemical parameters were analyzed in triplicate, and the results are presented as mean ± standard deviation (SD). Statistical analyses included the application of one-way ANOVA followed by Tukey’s HSD (Honestly Significant Difference) test and Student’s unpaired *t*-test [50]. Statistical significance was considered at *p* < 0.01. Cytotoxicity/antiproliferative activity were expressed as CC_50_/IC_50_ values (concentrations required for 50% cytotoxicity/antiproliferative activity), calculated using non-linear regression analysis. The statistical analysis included application of one-way ANOVA followed by Bonferroni’s post hoc test (GraphPad Prism 8 Software, San Diego, CA, USA). *p* < 0.05 was accepted as the lowest level of statistical significance. The data obtained are on average from three independent experiments ± SD, *n* = 6.

## 3. Results and Discussion

### 3.1. Chemical Composition and Physicochemical Characteristics of K. paniculata Seeds and Seed Oil

The contents of glyceride oil, proteins, carbohydrates, fiber, ash, and moisture in the seeds were determined (Table 1). *K. paniculata* seeds contain a high percentage of glyceride oil (over 20%). The total amount of carbohydrates (54.5%), among which starch was the best represented (14.2%), dominated over the proteins in the composition. The determined amounts of fiber (17.2%) characterize the seeds of *K. paniculata* as a good source of dietary fiber.

Other research have also characterized *K. paniculata* seeds and the oil obtained from them, regarding the chemical composition and physicochemical parameters. The oil content in *K. paniculata* seeds, found in our study, is close to the previous findings [51], who determined that the glyceride oil content of the seeds was 22.2%. On the other hand, some authors found an oil content in *K. paniculata* seeds over 40% [23]. Obviously, the method of glyceride oil isolation is important for the amount obtained (cold pressing, petroleum ether extraction, and hexane extraction) [25]. The author found that the highest yield was obtained with petroleum ether extraction (35.66%), while for cold-pressed seeds and those extracted with hexane, it was lower—25.51% and 32.34%, respectively. The yield of *K. paniculata* seed oil was close to that reported for the well-known olive oil (20%) and sesame oil (19%) [52,53]. The protein and fiber amounts, found in the seeds, were lower than those reported in the previous studies, 18.95–20.11% and 26.13–28.12%, respectively [23], and the ash and moisture contents were in agreement with the data published by [25]—3.53% and 6.90%. It was established that the seeds from *K. paniculata* were rich in glyceride oil and carbohydrates, especially starch and dietary fibers which made them a good source of these components. 

Seven physicochemical characteristics of glyceride oil were determined (Table 2). The peroxide value indicates the degree of oxidation of the vegetable oils, and the value for this indicator for the examined oil was 10 meq O_2_/kg. The acid value determined the free fatty acid content of the oils and the present value was in accordance with the requirements for glyceride oils (up to 4 mg KOH/g) [54]. The measured iodine value (44 gI_2_/100 g) characterizes *K. paniculata* seed oil as a non-drying oil. The oxidative stability of *K. paniculata* glyceride oil was over 50 h, indicating a high oxidative stability. The data for the other measured characteristics (saponification value, relative density, refractive index) are shown in Table 2.

The peroxide value of the tested glyceride oil from *K. paniculata* seeds was twice to almost five times higher than the values obtained by [25]. On the other hand, the acid value was lower compared to the previous studies—1.27 and 1.89 mg KOH/g [25,27,28]. The saponification value of the studied oil from *K. paniculata* was higher than previously established by [25] and [28], and those of the iodine value were almost twice lower, compared to the studies by other authors [25,27,28,51]. The refractive index of the glyceride oil coincides with the previously reported values [51]. Another study also concluded good oxidative stability, based on the measured peroxide value and p-anisidine value [25].

The content of fat-soluble biologically active components in the seeds and in the glyceride oil of the golden rain tree was also determined (Table 3). The content of unsaponifiable matter in *K. paniculata* seed oil was found to be 1.7% in the oil (0.3% in the seeds, respectively). The main representatives from this fraction were sterols (0.4% in the oil and 0.1% in the seeds) and tocopherols (345 mg/kg and 70 mg/kg in the oil and in the seeds, respectively). Total sterols were about 24% from the fraction of the unsaponifiables. According to [54], the total content of sterols in the studied glyceride oil approaches the values of some widely used vegetable oils, such as sunflower (0.24–0.46%), cotton (0.27–0.67%) and soybeans (0.18–0.41%). The total content of the phospholipids accounted to 2.2% in the oil and 0.4% in the seeds, respectively, which was similar to the total phospholipids in soybean oil (1.5–3.0%) and soybean seeds (0.3–0.6%) [55].

Eleven fatty acids were identified in *K. paniculata* glyceride oil composition (Table 4). The main components were eicosenoic acid (46.5%), followed by oleic acid (41.8%). The content of saturated palmitic acid was 6.6%, and that of polyunsaturated linoleic acid—3.4%. The amount of the rest fatty acids identified varied between 0.1 and 0.7%.

The percentage distribution of the different fatty acid classes in the oil composition is presented also in Table 4. Unsaturated fatty acids predominated in the oil (92.2%), with monounsaturated fatty acids being better represented than polyunsaturated fatty acids. The amount of saturated fatty acids in the lipid fraction was only 7.8%. The higher content of monounsaturated fatty acids is due to the greater amount of omega-9 oleic and eicosenoic acids in the oil.

High content of eicosenoic acid in the glyceride oil from the seeds of *K. paniculata* was also found in previous studies [24,25,27,28,51], where the amount varies between 43.3% and 48.5%. The same authors found that the content of oleic acid is from 21.77% to 32.0%, that of linoleic acid is from 7.01% to 13.0%, and that of palmitic acid is from 4.50% to 9.7%. Different results regarding the fatty acid composition of *K. paniculata* oil were published [26], who found that oleic acid is predominant (80.1%), and the content of the other fatty acids (palmitic, linoleic, stearic, and linoleic) is significantly low (respectively 8.0%, 6.7%, 4.0%, and 1.1%). The contents of saturated and unsaturated fatty acids in the glyceride oil from the studied *K. paniculata* seeds are in agreement with the data of previous studies, where unsaturated fatty acids predominate (from 84.1 to 88.5%), and that of saturated is between 11.5 and 15.9% [28]. Of the unsaturated fatty acids with a larger share were monounsaturated ones, the amount of which varied between 71.8 and 77.6% [25,28]. The consumption of foods rich in unsaturated fatty acids has many benefits for the human body—it improves physical functioning, accelerates metabolism, and provides protection against autoimmune diseases, cancer, osteoarthritis, etc. These compounds support cellular activities such as cellular signaling, cellular structural integrity, regulation of blood pressure, glucose levels, inflammatory responses, blood clotting, skeletal muscle metabolism, etc., [56]. Summary information from multiple sources reports that they improve the condition of inflammatory rheumatic diseases. When taken as food supplements, their intake leads to a significant reduction in pain, the number of swollen and sensitive joints, as well as a reduction in disease activity, compared to the control group. The consumption of unsaturated fatty acids can be an adjunctive therapy for the successful control of rheumatoid arthritis [57]. Many studies are being focused on the health benefits of MUFA and especially of oleic acid. There are numerous evidence on the impact of high oleic diet on the decreasing rate of the coronary heart disease, diabetes type 2, hypertension and may have protective effect against stroke [58,59,60]. A diet with high content of eicosenoic acid is also observed to have beneficial effect toward obesity-related metabolic dysfunctions [61].

The high percentage content of biologically active substances obtained in the glyceride oil became the reason for determining its individual sterol, tocopherol, and phospholipid composition. The data are presented in Table 5. Six phytosterols were identified and the main component in the sterol fraction was *β*-sitosterol (75.9%), followed by stigmasterol (15.1%) and campesterol (6.6%). A smaller amount of Δ^7^-stigmasterol-corresponding to 1.9% was found. The content of Δ^7^-campesterol and brassicasterol was significantly low. 

Three components were identified in the individual tocopherol composition of the studied glyceride oil, the main representative of which was *β*-tocopherol (56.6% of the total tocopherol content), followed by *γ*-tocopherol (33.4%) and *α*-tocopherol (10.0%) (Table 5). Six phospholipid classes were identified, which were distributed between 29.1% and 12.8%. The highest amount was phosphatidylcholine (29.1%), followed by phosphatidylinositol (17.5%), and the remaining phospholipids were represented in almost equal amounts—from 12.8% (diphosphatidylglycerol) to 14.2% (phosphatidylethanolamine). The individual phospholipid composition of *K. paniculata* seeds was determined for the first time in the present study. Vitamin E, sitosterol, and stigmasterol were also chromatographically identified in extracts of *K. paniculata* plant parts [13,14,15,16]. 

Phytosterols are a group of bioorganic molecules known for a long history of consumption as food and pharmaceutical products [62]. *β*-sitosterol, which was represented in the highest percentage content of the studied group of compounds, is one of the most abundant, naturally occurring phytosterols in plants. In the review of the above-cited authors, *β*-sitosterol has therapeutic value against various tumor cells (malignant tumors of the prostate, breast, kidney, pancreas, stomach, lung, and other cancers), confirmed by various pharmacological studies. Some of its antitumor mechanisms are indicated, which enables its use in the preparation of new antitumor drugs [62]. *β*-sitosterol is contained in various dietary and non-dietary plants. In vivo studies on mice proved its safety—it does not cause genotoxicity and cytotoxicity [63]. In addition to being proven harmless, the authors report various beneficial health effects—it reduces the risk of coronary disease, heart attack, and atherosclerosis; it lowers the level of low density lipid plasma cholesterol (LDL), as well as supports the body’s natural recovery process. Other studies have shown the strong anti-inflammatory activity of *β*-sitosterol derived from plant extracts, accounted for by the release of histamine, serotonin, bradykinin, and prostaglandin [64]. 

Total tocopherol content of the examined oil from *K. paniculata* seeds was similar to those of grape seed oil (240–410 mg/kg), safflower oil (240–670 mg/kg) [54], and amaranth seed oil (54.2–55.5%) [65]. The three isoforms of vitamin E (*α-*, *β-*, *γ*-tocopherol) in *K. paniculata* seed oil, proven in our study, represent a part of the eight isoforms, in which this vitamin exists. It has been found that some isoforms suppress the generation of new free radicals (*α*-tocopherol), and others neutralize the existing ones (*γ*-tocopherol) [66]. Vitamin E is an essential antioxidant in cardiovascular disease prevention [67] and it plays a protective role in a number of diseases resulting from the action of free radicals—atherosclerosis, cancer, Alzheimer’s, etc., [66]. 

Phospholipids are another well-represented bio-active group of the lipid fraction. They are the main components of cell membranes and they have various physiological properties that determine their pharmaceutical potential [68]. According to recent research, their ability to form liposomes makes them suitable carrier molecules for medicinal compounds. They can increasingly penetrate through the skin and, when applied locally, enhance the effectiveness of the active components. In addition, the formed phospholipid complexes are more stable compared to other lipids, which guarantees their stability and facilitates the preparation of substances for the treatment of various diseases [69].

### 3.2. Qualitative In Vitro DNA-Protective Assay 

**Figure 2:**
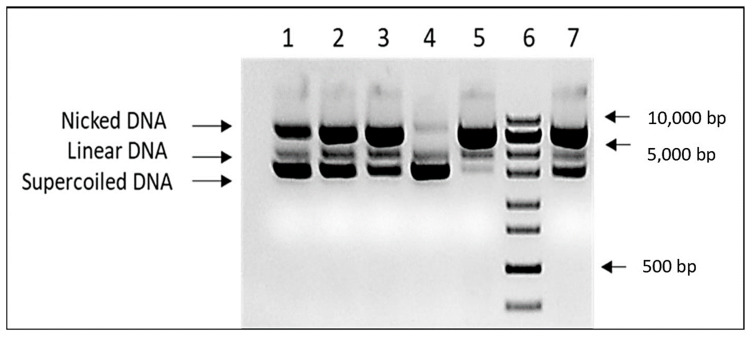
DNA protective activity of *K. paniculata* glyceride oil. Lines 1–3—different concentrations of Trolox (25, 50, and 100 mg/mL), line 4—pUC19 input, line 5-negative (water) control, line 6—ZipRuler 1 Express DNA Ladder (Thermo Scientific, Waltham MA USA, cat № SM1373), and line 7—*K. paniculata* glyceride oil.

The DNA protective capacity of the *K. paniculata* glyceride oil was assessed in the in vitro DNA nicking assay experiment. In order to overcome the oil solubility issue, DNA protective assay was performed in the presence of cyclodextrin at a final concentration of 6% in the assay mixture. The results are shown in Figure 2. The presence of 1 µL of the tested oil significantly reduces the amount of nicked DNA. Due to the limited extract solubility in aqueous solutions, it was impossible to demonstrate concentration-dependent DNA protection. Unlike in all Trolox controls, a clear correlation between DNA damage and antioxidant concentration was found. 

The results obtained by the molecular genetic analysis confirm the protective role against DNA nicking for another plant product from the species, which is its glyceride oil. The current DNA test is a continuation of our previous studies in which we found DNA protective activity against generated free radicals (H_2_O_2_) when ethanol extracts (from the aerial plant parts) of *K. paniculata* are applied [20]. The effect observed in the present study is consistent with the gene-protective activity of methanol leaf extracts from *K. paniculata* as reported by [4]. Moreover, leaf extracts (and hexane fraction) effectively remove H_2_O_2_-induced damage to PUC18/calf thymus DNA [17], as well as those caused by 4-nitroquinoline-1-oxide [19]. The ethyl acetate and chloroform leaf fractions from the golden rain tree contribute to the removal of DNA damage induced by Fenton’s reagent in pUC18 plasmid DNA [18]. 

### 3.3. Cytotoxicity and Antiproliferative Activities

**Figure 3:**
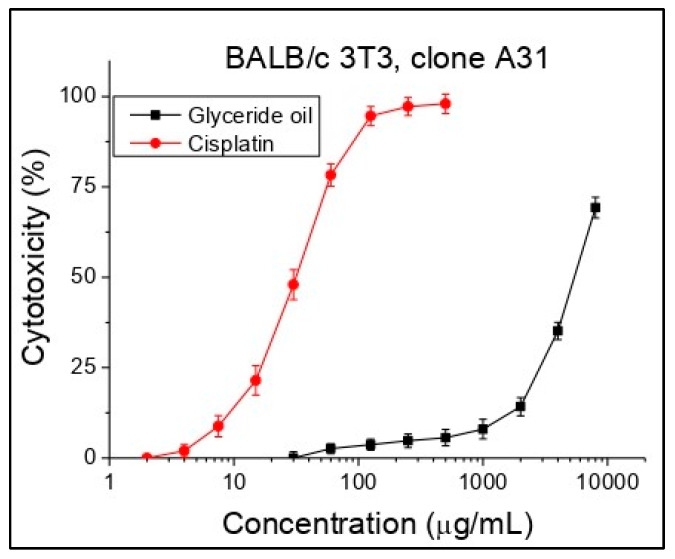
Cytotoxicity of *K. paniculata* glyceride oil determined in the cell line BALB/c 3T3 clone A31, *n* = 6.

Cytotoxicity was expressed as CC_50_ (50% cytotoxic concentration) values oil concentrations (μg/mL). Cisplatin was used as a standard in this investigation. Concentrations from 30 to 8000 µg/mL were tested. Based on the obtained sigmoidal curves, the average CC_50_ values (mean cytotoxic concentrations) for each investigated substance were calculated (Figure 3, Table 6). While for the standard cisplatin initial cytotoxicity was observed at a concentration of about 7.5 µg/mL, for the glyceride oil, an initial effect was established at a higher concentration (after 1000 µg/mL), which indicates a lack of cytotoxicity. As can be seen from the table, the glyceride oil showed more than 130 times less cytotoxicity compared to the standard in the study of cisplatin, which practically means no cytotoxicity against the cell line BALB/c 3T3, clone A31.

**Figure 4:**
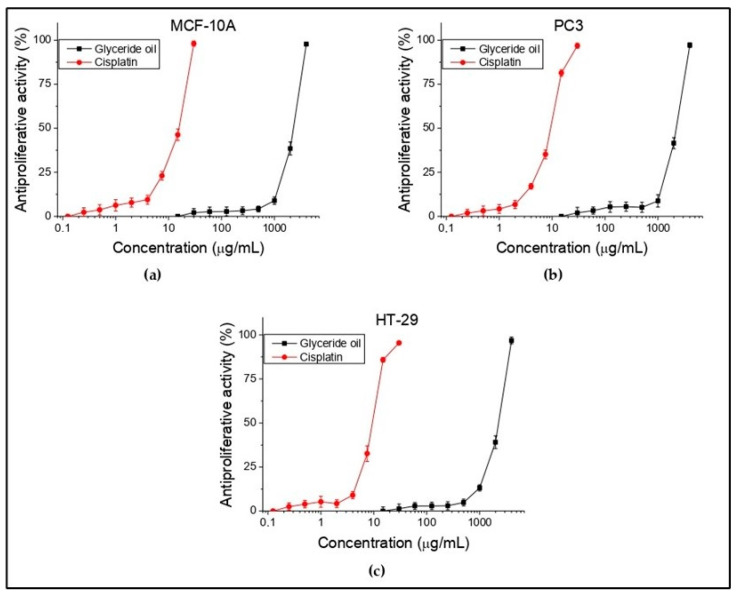
In vitro antiproliferative activity (%) of *K. paniculata* glyceride oil on normal human (**a**) and tumor (**b**,**c**) cell lines. *n* = 6.

In the present study, in vitro antiproliferative test were conducted on HT-29 (colorectal adenocarcinoma) and PC3 (prostate carcinoma) tumor cell lines and MCF-10A (normal human epithelial cells). Antiproliferative activity was expressed as IC50 values (oil concentrations (μg/mL) required for 50% inhibition of cell growth). Cisplatin was used again as a standard to confirm the suitability of the used antitumor method. The obtained results are summarized and shown in Figure 4 and Table 7. Concentrations from 15 to 4000 µg/mL for the oil were tested. It is apparent that there is no activity up to 10 µg/mL, which is for all three cell lines. Such is observed at concentration above 1000 µg/mL. The selectivity (SI) is ratio of the investigated substance to normal and to tumor cell line. The higher index value means higher selectivity. In the present study, this was not reported in both the investigated cell lines. The obtained results showed that the tested oil did not show antiproliferative activity against the tested tumor and normal human cell lines. Moreover, no selectivity toward them was found.

The information on the studied antitumor activity in the species is scarce, so a comparison cannot be made. Week antineoplastic potential on human hepatocarcinoma (HepG2) and human breast cancer cells (MDA-MB-231) is reported for pure carotenoid fraction, isolated from flowers [21]. In our previous study, a significant antiproliferative activity on the HT-29 cell line was found for the flower and leaf ethanol extracts of *K. paniculata* and a weaker one for bark extract [22]. The study showed that extracts from the different plant parts of the tree species had an antitumor potential, but no such effect was confirmed for the glyceride seed oil. Other tumor cell lines could be tested in future studies.

## 4. Conclusions

The evaluation of the chemical composition of *K. paniculata* seeds showed that they are rich in glyceride oil, dietary fiber, protein, and starch. Glyceride oil has high oxidant stability, it is rich in healthy unsaturated fatty acids (mainly omega-9 eicosenoic and oleic acids), vitamin E (*α*-, *β*-, and *γ*-tocopherols), sterols (*β*-sitosterol prevail), and for the first time established phospholipids (phosphatidylcholine was the major compound). DNA protective activity of glyceride oil was proven for the first time by using a qualitative in vitro DNA nicking protection assay, as well as a lack of cytotoxicity. Unfortunately, there was not found any antiproliferative activity of the oil against PC3 and HT-29 human tumor cell lines. In brief, *K. paniculata* glyceride oil could be a source of health-promoting components or a natural preservative in various food, pharmaceutical, and cosmetic products. 

## Figures and Tables

**Table 1:** Chemical composition of *K. paniculata* seeds.

Parameter	Content (%) *
Oil content	20.4 ± 0.3
Proteins	15.1 ± 0.2
Carbohydrates	54.5 ± 0.7
» starch	14.2 ± 0.1
» reducing sugars	0.48 ± 0.04
» invert sugar	2.66 ± 0.16
» fiber	17.2 ± 0.2
Ash	3.3 ± 0.1
Moisture	6.7 ± 0.1

* The samples were analyzed in triplicate, and the results were expressed as mean ± standard deviation. »—the components noted are part of the total carbohydrates.

**Table 2:** Physicochemical characteristics of the glyceride oil.

Parameter	Content *
Peroxide value, meq O_2_/kg	10.0 ± 0.1
Acid value, mg KOH/g	0.8 ± 0.0
Iodine value, gI_2_/100 g	44 ± 0.2
Saponification value, mg KOH/g	209 ± 1
Relative density	0.9 ± 0.0003
Refractive index	1.5 ± 0.0002
Oxidative stability, h	Over 50

* The sample was analyzed in triplicate, and the results were expressed as mean ± standard deviation.

**Table 3:** Biologically active components in *K. paniculata* seeds and seed oil.

	Biologically Active Components			
	Unsaponifiable Matter, %	Phospholipids, %	Sterols, %	Tocopherols, mg/kg
-in the oil	1.7 ± 0.1 ^a^	2.2 ± 0.1 ^a^	0.4 ± 0.0 ^a^	345 ± 8 ^a^
-in the seeds	0.3 ± 0.0 ^b^	0.4 ± 0.0 ^b^	0.1 ± 0.0 ^b^	70 ± 2 ^b^

The samples were analyzed in triplicate, and the results were expressed as mean ± standard deviation. Different letters next to the values in the same column mean significant differences (*p* < 0.01) using Student’s *t*-test.

**Table 4:** Fatty acid composition of the seed oil.

Fatty Acids *	Content ** (%)
C _14:0_	0.1 ± 0.0
C _14:1_	0.2 ± 0.0
C _16:0_	6.6 ± 0.1
C _16:1_	0.1 ± 0.0
C _17:1_	0.1 ± 0.0
C _18:0_	0.4 ± 0.1
C _18:1_	41.8 ± 0.2
C _18:2_	3.4 ± 0.0
C _20:1_	46.5 ± 0.2
C _20:5_	0.1 ± 0.0
C _23:0_	0.7 ± 0.0
SFA	7.8
MUFA	88.7
PUFA	3.5

* C _14:0_—myristic; C _14:1_—myristoleic; C _16:0_—palmitic; C _16:1_—palmitoleic; C _17:1_—heptadecenoic; C _18:0_—stearic; C _18:1_—oleic; C _18:2_—linoleic; C _20:1_—eicosenoic; C _20:5_—eicosapentaenoic; C _23:0_—tricosanoic; SFA—saturated fatty acids; MUFA—monounsaturated fatty acids; PUFA—polyunsaturated fatty acids. ** The sample was analyzed in triplicate, and the results were expressed as mean ± standard deviation.

**Table 5:** Composition of sterols, tocopherols, and phospholipids in the glyceride oil.

Sterols, %		Tocopherols, %		Phospholipids, %	
Cholesterol	n.d. *	*α*-Tocopherol	10.0 ± 0.1 ^c^	Phosphatidic acids	13.4 ± 0.2 ^d^
Brassicasterol	0.2 ± 0.0 ^e^	*β*-Tocopherol	56.6 ± 0.2 ^a^	Phosphatidylserine	13.0 ± 0.2 ^d^
Campesterol	6.6 ± 0.1 ^c^	*γ*-Tocopherol	33.4 ± 0.1 ^b^	Phosphatidylinositol	17.5 ± 0.1 ^b^
Stigmasterol	15.1 ± 0.1 ^b^			Phosphatidylcholine	29.1 ± 0.3 ^a^
Δ^7^-Campesterol	0.3 ± 0.0 ^e^			Phosphatidylethanolamine	14.2 ± 0.1 ^c^
β-Sitosterol	75.9 ± 0.2 ^a^			Diphosphatidylglycerol	12.8 ± 0.1 ^d^
Δ^7^-Stigmasterol	1.9 ± 0.1 ^d^				

The samples were analyzed in triplicate, and the results were expressed as mean ± standard deviation. According to Tukey’s HSD test (*p* < 0.01), different superscript letters denote significant differences. *—not detected.

**Table 6:** Cytotoxicity in BALB/c 3T3, CC_50_ values.

Compounds	Mean CC_50_ ± SD (µg/mL)
Glyceride oil	5402.24 ± 121.01
Cisplatin	31.35 ± 2.72

The samples were analyzed in triplicate, and the results were expressed as mean ± standard deviation.

**Table 7:** In vitro antiproliferative activity (IC_50_ values) of *K. paniculata* glyceride oil.

Compounds	Mean IC_50_ ± SD (µg/mL)			SI *	
	MCF-10A	PC3	HT-29	PC3	HT-29
Glyceride oil	2284.76 ± 79.14	2221 ± 79.74	2274.50 ± 79.77	1.03	1
Cisplatin	15.73 ± 0.63	9.38 ± 0.27	9.4 ±0.4	1.68	1.67

* Selective index (SI)-IC_50_ determined following 72 h treatment with glyceride oil. The samples were analyzed in triplicate, and the results were expressed as mean ± standard deviation.

## Data Availability

Data is contained within the article.
